# Supplementary material for: Eating Patterns among Emergency Medical Service Providers in the United States: A Qualitative Interview Study
Source: Nutrients. 2022 Nov 18;14(22):4884. doi: 10.3390/nu14224884 (PMC9696925; doi:10.3390/nu14224884)
Supplement: Supplementary file 1 [file nutrients-14-04884-s001.zip › nutrients-2002419-supplementary.pdf]

Supplementary Table S1. Interview guide for semi-structured interviews with 40 EMS providers throughout the United States regarding sleep and eating patterns. The interview questions were determined based on research questions. Probes such as "can you explain what you mean by that" and "can you tell me more about that" were used to gain further insights on topics of discussion.

Research question	Interview questions	Follow up questions
What factors specific to EMS influence eating patterns?	How would you describe your food choice process on shift?	What other factors influence what you eat during a shift?
	How does your work environment influence your food choice?	Can you describe what you mean?
	How does your partner influence your food choice?	How so? Positive/negative? Does anyone else at work influence your food choice?
	How does your stress level influence how you eat on and off your shift?	What influences that? How often does this occur?
	How does eating/what you eat affect how you feel at work?	How so? How does this affect your food choice?
What factors specific to EMS affect sleep patterns?	What else influences your sleep patterns?	How many hours/night on average do you sleep? How consistent?
	How would you describe the quality of your sleep?	What influences that?
How do days off differ from shift days among providers?	How do your eating patterns differ?	What influences your food choice at home?
	How do your sleep patterns differ? Are you able to recover from your shift?	If no, why not?
What are barriers to changing sleep and eating patterns exist specific to the EMS lifestyle?	What, if any, eating related goals do you have?	What influences this?
	What affects the quality of your sleep?	Can you describe more?
What methods have been adopted by EMS providers to make the job more sustainable?	What challenges do you face with eating at work, and how do you overcome them?	How does this impact you during a shift?
	What coping skills help you recover from challenging calls?	How does that affect you?
